# Raised Plasma Robo4 and Cardiac Surgery-Associated Acute Kidney Injury

**DOI:** 10.1371/journal.pone.0111459

**Published:** 2014-10-31

**Authors:** Anne Burke-Gaffney, Tatiana Svermova, Sharon Mumby, Simon J. Finney, Timothy W. Evans

**Affiliations:** 1 Vascular Biology, Cardiovascular Sciences, National Heart and Lung Institute Division, Faculty of Medicine, Imperial College London, London, United Kingdom; 2 National Institute for Health Research Respiratory Biomedical Research Unit, Royal Brompton and Harefield NHS Foundation Trust, London, United Kingdom; Northwestern Polytechnical University, China

## Abstract

**Objective:**

Endothelial dysfunction associated with systemic inflammation can contribute to organ injury/failure following cardiac surgery requiring cardiopulmonary bypass (CPB). Roundabout protein 4 (Robo4), an endothelial-expressed transmembrane receptor and regulator of cell activation, is an important inhibitor of endothelial hyper-permeability. We investigated the hypothesis that plasma levels of Robo4 are indicative of organ injury, in particular acute kidney injury (AKI), after cardiac surgery.

**Methods:**

Patients (n = 32) undergoing elective cardiac surgery with CPB were enrolled, prospectively. Plasma Robo4 concentrations were measured pre-, 2 and 24 h post-operatively, using a commercially available ELISA. Plasma and endothelial markers of inflammation [interleukin (IL) -6, -8, -10: von Willibrand factor (vWF) and angiopoeitin-2 (Ang-2)] and the AKI marker, neutrophil gelatinase-associated lipocalin (NGAL), were also measured by ELISA.

**Results:**

Plasma Robo4 increased significantly (p<0.001) from pre-operative levels of 2515±904 pg/ml to 4473±1915 pg/ml, 2 h after surgery; and returned to basal levels (2682±979 pg/ml) by 24 h. Plasma cytokines, vWF and NGAL also increased 2 h post-operatively and remained elevated at 24 h. Ang-2 increased 24 h post-operatively, only. There was a positive, significant correlation (r = 0.385, p = 0.0298) between Robo-4 and IL-10, but not other cytokines, 2 h post-operatively. Whilst raised Robo4 did not correlate with indices of lung dysfunction or other biomarkers of endothelial activation; there was a positive, significant correlation between raised (2 h) plasma NGAL and Robo4 (r = 0.4322, p = 0.0135). When patients were classed as AKI or non-AKI either using NGAL cut-off of 150 ng/ml, or the AKI Network (AKIN) clinical classification; plasma Robo4 was significantly higher (p = 0.0073 and 0.003, respectively) in AKI vs. non-AKI patients (NGAL cut-off: 5350±2191 ng/ml, n = 16 vs. 3595±1068 pg/ml, n = 16; AKIN: 6546 pg/ml, IQR 5025–8079, n = 6; vs. 3727 pg/ml, IQR 1962–3727, n = 26) subjects.

**Conclusion:**

Plasma Robo4 levels are increased, transiently, following cardiac surgery requiring CPB; and higher levels in patients with AKI suggest a link between endothelial dysregulation and onset of AKI.

## Introduction

Activation of the vascular endothelium occurs following cardiac surgery requiring cardiopulmonary bypass (CPB) [1]; and contributes to the systemic inflammatory response (SIRS) that manifest in the majority of such patients post-operatively [2]. In a highly regulated sequence of events, encompassing increased coagulant and adhesive states, release of pro-inflammatory mediators and loss of barrier function; extravasation of excess fluid leads to tissue oedema and eventually organ dysfunction. Devastating organ injuries such as acute lung injury (ALI) [3] and acute kidney injury (AKI) [4], [5], are amongst the most frequent complications of cardiac surgery with CPB. Endothelial biomarkers are not measured routinely in the context of cardiac surgery requiring CPB, but we hypothesised that such measurements might assist in identifying those most at risk of developing organ failure and subsequent morbidity and mortality.

SIRS [6], sepsis [7] and infectious diseases [8], are associated with perturbations in endothelial markers including those relevant to coagulation, adhesion, vascular tone and permeability. Of these, von Willebrand factor (vWF) and angiopoeitin-2 (Ang2) have been the most extensively studied and showed associations with organ dysfunction, notably ALI [6], [8]. Further, a doubling in plasma vWF that was positively associated with urinary albumin creatinine ratio was reported following CPB, linking endothelial injury with increased permeability [9]. More recently, another study also showed increased vWF after CPB, but associations with other indices were not sought [10]. Raised Ang-2 levels after CPB, in children, correlated with intensive care unit length of stay (ICU LOS) ([11]; and in adults, with duration of mechanical ventilation, ICU LOS and mortality [12]).

Roundabout (Robo) proteins are a family of transmembrane receptors originally identified for their role in axon guidance during development of the nervous system [13]. Four receptors have been characterised, of which Robo-1, -2 and -3 share a common extracellular structure similar to adhesion molecules; with five immunoglobulin-like (Ig) domains and three fibronectin type 3 (FN3) repeats [14], [15]. The fourth, Robo4 or magic roundabout protein, is found only in vertebrates and has 2 Ig domains and 2 FN3 repeats [14], [15]. Following initial identification as an endothelial expressed gene [16], Robo4 was designated a tumour endothelial marker and its role in endothelial migration determined [17]. It has since been detected in vascular smooth muscle [18], hematopoietic stem [19] and rat airway smooth muscle [20] cells, with functional roles in cell migration also described.

Robo4 is an important inhibitor of pathologic angiogenesis and endothelial hyper-permeability, that prevented VEGF-induced changes in models of retinal and choroidal vascular disease [21], [22]; and hyper-permeability in bacterial and viral models of sepsis [23]. Robo4 interaction with its ligand Slit2 also prevented pathological angiogenesis in virus-induced keratitis [24]; HIV-induced lymphatic hyper-permeability [25]; changes in retinal endothelial permeability [26]; Andes virus-induced permeability increases in lung microvascular endothelial cells [27]; and inhibition of LPS-induced cytokine/chemokine release and ICAM-1 expression in HUVEC [28]. Also, a novel anti-inflammatory strategy (apoA-I mimetic 4F) that increased Robo4/Slit2 expression strengthened vascular barrier function, protecting kidneys and heart in an animal model of sepsis [29].

To our knowledge, no other study has yet quantified plasma Robo4 in clinical SIRS. We therefore explored the hypotheses: first, that Robo4 would be detected and elevated in plasma of patients following CPB. Second, that raised Robo4 levels would correlate with changes in established markers of systemic inflammation and endothelial activation; and finally, that increases in Robo4 would correspond with markers of organ failure, proving a useful early predictor of those most at risk of complications, post cardiac surgery requiring CPB. Indeed, our findings showed that plasma levels of Robo4 levels were significantly elevated, albeit transiently, post CPB and were higher in patients deemed to have AKI.

## Methods

### Patient population and sample collection

Samples and clinical data were collected from thirty-two patients recruited sequentially at the Royal Brompton Hospital, London, UK (December 2008 to August 2009) undergoing surgery for valve replacement and/or coronary artery bypass grafting (CABG) and requiring CPB. Exclusion criteria were lack of informed consent. Blood was collected via indwelling cannulæ; and if not for immediate analysis, processed and plasma samples stored at −80°C. Samples were collected immediately prior to surgery and at 0, 2, 4, 6 and 24 h post-operatively.

### Robo4 and inflammatory/endothelial marker assays

Commercially available sandwich enzyme-linked immunosorbent assay (ELISA) kits were used to measure: Robo4 (Cusabio Biotech, Suffolk, UK); IL-6, -8 (Hycult Biotechnology, Uden, The Netherlands); IL-10 (BD Bioscience, Oxford, UK); NGAL(BioPorto Diagnostics A/S, Hellerup, Denmark); Ang-2 (R&D Systems, Oxford, U.K.); and vWF (Zymutest vWF kit; Hyphen-Biomed, Neuville-sur-Oise, France). Biomarkers were measured at all the time points in: all patients (cytokines/NGAL); or a pilot study of 3 patients (Robo4, vWF and Ang-2); and subsequently, pre-, 2 and 24 h post-operatively- a choice dictated by pilot study data and sample availability.

### Clinical data

Pre-operative characteristics, age, gender, Body Mass Index (BMI), creatinine and a widely employed clinical risk assessment tool (additive EUROscore) were recorded. Operative (nature of procedure; and duration from beginning of anaesthesia to end of CPB) and post-operative variables were obtained following interrogation of an automated clinical data collection system; and included post-operative oxygenation (PaO_2_:FiO_2_ [PF] ratio), as an indicator of pulmonary dysfunction (first value recorded on return to ICU); and duration of mechanical ventilation. Patients were defined as having AKI or not (non-AKI), using NGAL cut-off levels of <or>150 ng/ml [30]; and secondly, using the AKI Network (AKIN) classification system [4]. Indices of clinical outcome included duration of level 3 dependency, and ICU and hospital LOS [2], [31]. The former was employed as a composite index reflecting the degree of physiological derangement and the requirements for supportive care. The study protocol was approved by the Research Ethics Committee for the Royal Brompton and Harefield NHS Foundation Trust and informed written consent was obtained from all patients.

### Statistics

Statistical analyses were performed to establish how plasma biomarkers were altered following surgery and to investigate associations with clinical indices. Non-parametric data were expressed as median and interquartile range (IQR) and compared using: Wilcoxon matched-pairs test with Dunn’s correction for 2 paired groups and Friedman's test for 3 paired groups; or Mann-Whitney U test for 2 unpaired groups. Parametric data were expressed as mean ± standard deviation (SD) and compared using: repeated measures ANOVA and Tukey's Multiple Comparison Test; or Student’s t test for unpaired data. Correlations between variables were assessed using Spearman analysis (non-parametric, stating r) or Pearson’s parametric analysis (stating r and r^2^), with associated p values. P values of <0.05 were considered significant. Excel 2007 (Microsoft, CA) and Prism v5.04 (Graphpad software, CA) were used for analyses.

## Results

### Patient characteristics

Demographics, clinical characteristics, operative variables and post-operative outcomes of the patient population (n = 32) are shown in Table 1. Median duration of level 3 care required was 33.7 h (IQR, 18.4–69); ICU LOS was 44.3 h (IQR, 23–56.4); and hospital LOS, 10 days (IQR, 9–12.5). Overall hospital mortality was 0%.

**Table 1:** Clinical and biochemical characteristics, operative variables and post-operative outcomes of study patients^a^.

Pre-operative characteristics	
Age, mean (SD) years	66 (10)
Gender F/M	7/25
Body Mass Index, median (IQR) kg/m^2^	26.9 (25.1–28.95)
Creatinine µmol/L, mean (SD)	93.22 (24.96)
**Operative variables**	
Operation	
CABG alone, n (%)	12 (37.5)
Valve repair/replacement, n (%)	15 (46.9)
CABG plus valve surgery, n (%)	15 (46.9)
Duration from start of anaesthesia to endbypass, median (IQR) min	216.5 (179–267)
**Post-operative outcomes**	
ICU length of stay, median (IQR) h	44.3 (23–56.4)
Level 3 care duration, median (IQR) h	33.7 (18.4–69)
Hospital length of stay, median (IQR) days	10 (9–12.5)
Hospital mortality, n (%)	0 (0)

a n = 32 patients undergoing cardiac surgery requiring cardiopulmonary bypass.

IQR = interquartile range; SD = standard deviation; CABG = Coronary artery bypass grafting; ICU = intensive care unit.

### Plasma levels of Robo4 increased post-cardiac surgery requiring CPB

**Figure 1:**
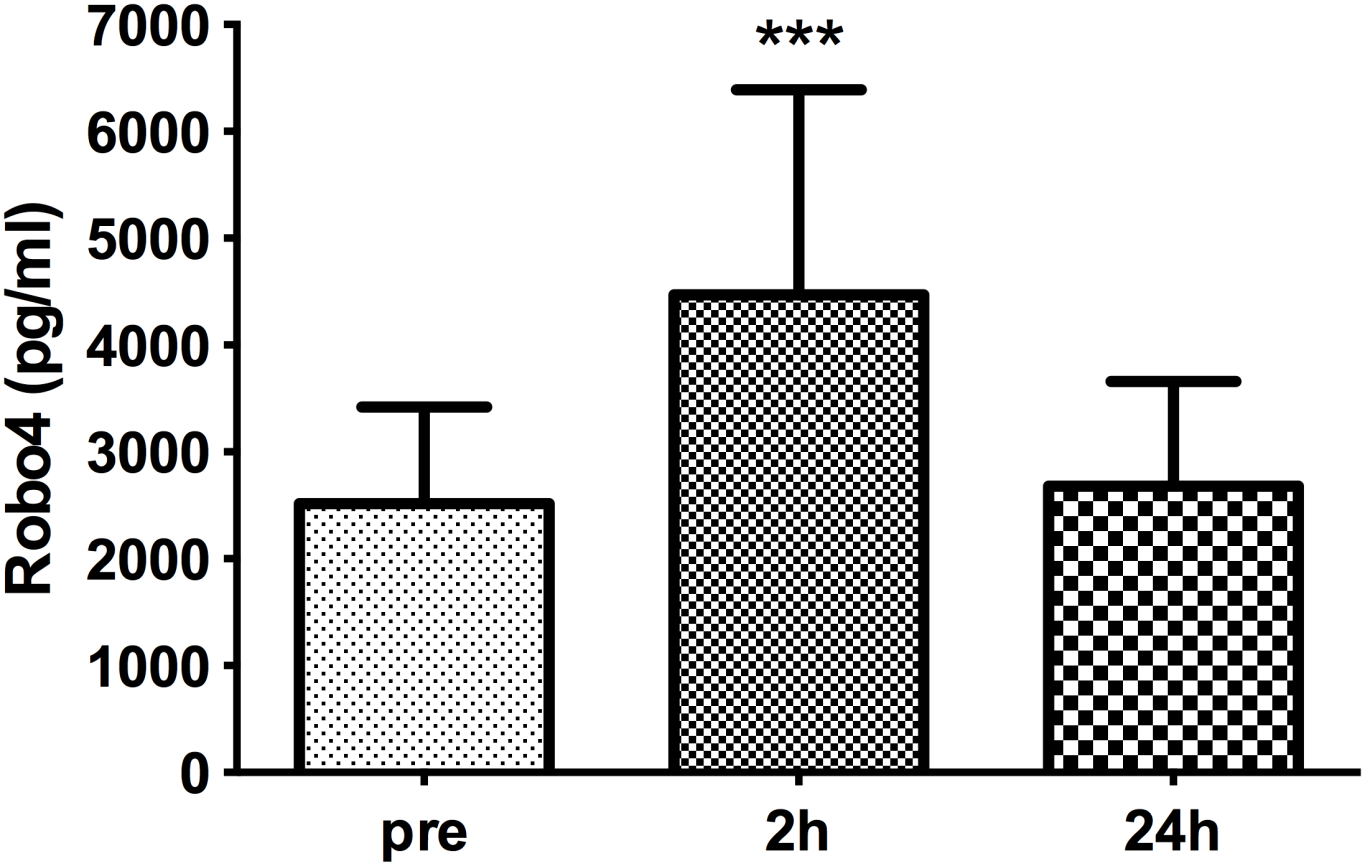
Plasma levels of Robo4 before and after cardiac surgery. Plasma levels of Robo4 increased 2 h post-cardiac surgery requiring cardiopulmonary bypass compared with pre-operative levels and those 24 h post-surgery. Data are presented as bar graphs with mean and SD concentrations (pg/ml) of Robo4; n = 32, ***p<0.001 compared with pre- and 24 h levels.

Plasma levels of Robo4 were significantly (p<0.001) higher in patients 2 h post-operatively (4473±1915 pg/ml) compared with levels in paired samples taken prior to surgery (2515±904 pg/ml); returning to basal levels by 24 h (2682±979 pg/ml; Fig. 1).

Utilising the patient characteristics in Table 1, significant, positive correlations were found between pre-operative levels of Robo4 with BMI (r = 0.37, p = 0.038) and EUROScore (r = 0.413, r^2^ = 0.1702, p = 0.019). However, there were no significant differences when patients were divided either into normal, overweight and obese groups (data not shown); nor of EUROscore ≥6 (high risk; 2778±724 pg/ml, n = 16) vs <6 (2253±1008 pg/ml, n = 16). When operative variables were considered, no associations were found with duration of operation (defined here as induction of anaesthesia to end of CPB). Robo4 levels did not vary significantly depending on the operative procedure; nor were associations found with ICU LOS, duration of level 3 Care, or hospital LOS (data not shown).

### Indices of systemic inflammation increased post cardiac surgery requiring CPB

We have previously utilised raised CRP and IL-8 levels to confirm post-operative systemic inflammation [31]. Pre-operative CRP was normal but increased significantly (p<0.0001) by day 1, post-operatively; as did white cell count (p = 0.0378), but the latter remained within the normal range (Table 2). Cytokine biomarkers of systemic inflammation showed characteristic plasma profiles with time; increasing significantly at 2 h and remaining elevated, albeit at lower levels, 24 h post-operatively (Table 2). Of the cytokines measured, there was a moderate significant positive correlation between IL-10 and Robo4 levels, 2 h post-operatively (r = 0.385, p = 0.0298).

**Table 2:** Indices of systemic inflammation^a^.

	Pre-op	2 h post-op	24 h post-op	Post-op day 1
**CRP**				
median (IQR) mg/L	2 (1–4)			54 (39.5–70)**^†^**
**WCC mean**				
mean (SD), 10^3^/µl	7.3±1.8			8.2±2.3*
**IL-6**				
median (IQR), pg/ml	19.7 (6.9–35.0)	101.5 (52.9–244)***	61.1 (33.3–173.2)***	
**IL-8**				
median (IQR) pg/ml	4.4 (2.2–6.5)	55.5 (30.3–21)***	12.7 (6.4–23.2)** ^,^ #	
**IL-10**				
median (IQR) pg/ml	4.8 (2.3–15.7)	63.7 (40.5–110)***	32.2 (21.6–47.4)*** ^,^ #	

a n = 32 patients undergoing cardiac surgery requiring cardiopulmonary bypass.

CRP, c-reactive protein; WCC, white cell count; IL-6, -8,-10, interleukin-6, -8,-10; IQR = interquartile range, SD = standard deviation.

*p = 0.0378, **p<0.01, ***p<0.001, **^†^**p<0.0001, compared with pre-op levels.

# p<0.05, compared with 2 h.

### Indices of organ dysfunction/injury following cardiac surgery requiring CPB

Baseline values of accepted biomarkers of kidney function (plasma and urine NGAL) and serum creatinine were within normal range (Table 3). Plasma and urine NGAL levels were significantly increased (p<0.001) at 2 and 24 h (Table 3). Whilst there was no association between creatinine (pre or post-operatively) and Robo4 levels, there were positive associations between plasma NGAL and Robo4 levels pre- (r = 0.3937, p = 0.0258) and 2 h post- (r = 0.4322, p = 0.0135) operatively. There was also a strongly positive, significant association between urine NGAL levels and plasma Robo4 at 2 h (r = 0.6998, p<0.0001).

**Table 3:** Indices of organ dysfunction/injury^a^.

	Pre-op	2 h post-op	24 h post-op	Post-op day 1
**Kidney**				
**NGAL (plasma)**	48.43	150.6	120.2	
median (IQR), ng/ml	(30.9–65.2)	(109.7–193.4)***	(92.68–180.9)***	
**NGAL (urine)**	5.07	15.57	27.95	
median (IQR), ng/ml	(2.55–11.63)	(6.58–123.7)*** ^b^	(17.65–70.67)***	
**Creatinine**	89.5			86.5
µmol/L median (IQR)	(75–111)			(76–103.5)
**Endothelium**				
**vWF**	87.16	136.6	152.9	
mean (SD), %	(26.8)	(55.2)***	(60.5)***	
**Ang-2**	2.32	2.54	7.314	
median (IQR), ng/ml	(1.89–3.59)	(1.92–3.71)	(5.38–9.55)*** ^,###^	

a n = 32 patients undergoing cardiac surgery requiring cardiopulmonary bypass; ^b^n = 31.

NGAL, neutrophil gelatinase-associated lipocalin; vWF, von Willibrand Factor; Ang-2, Angiopoeitin-2; IQR = interquartile range, SD = standard deviation.

***p<0.001, compared with pre-op levels; ^###^p<0.001 compared with 2 h.

**Figure 2:**
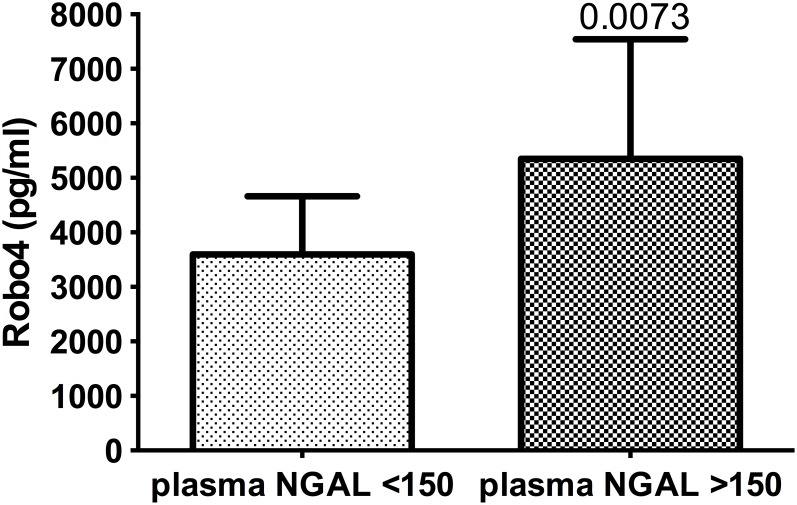
Plasma Robo4 levels in patients with or without AKI based on NGAL levels. Plasma levels of Robo4 in patients, 2 h post-cardiac surgery requiring cardiopulmonary bypass with or without acute kidney injury (AKI); assigned using plasma NGAL cut-off levels of 150 ng/ml. Data presented as bar graphs with mean and SD concentrations (pg/ml) of Robo4; n = 16 (AKI), n = 16 (non-AKI).

**Figure 3:**
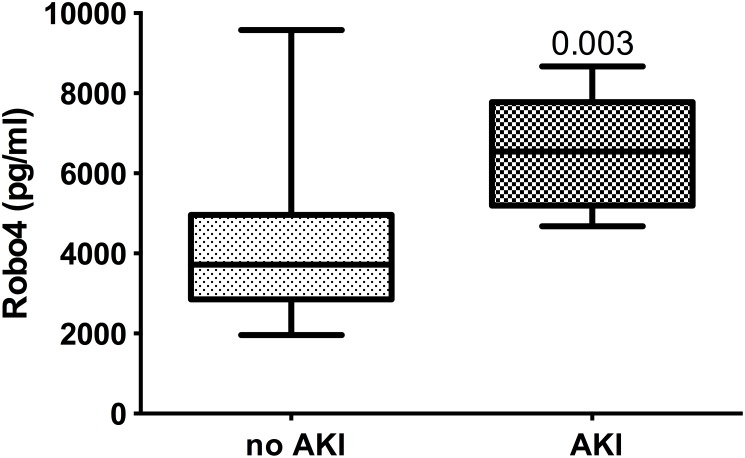
Plasma Robo4 levels in patients with or without AKI according to AKI Network classification. Plasma levels of Robo4 in patients, 2 h post-cardiac surgery requiring cardiopulmonary bypass with or without acute kidney injury (AKI); assigned according to AKI Network clinical classification. Data presented as median, IQR concentrations (pg/ml) of Robo4 (n = 26, non-AKI and n = 6, AKI).

Robo4 was significantly (p = 0.0073) greater in the patient group with plasma levels of NGAL of >150 ng/ml (5350±2191 ng/ml, n = 16), than in the group with NGAL levels <150 ng/ml (3595±1068 pg/ml, n = 16; Fig. 2). Likewise, when grouped based on urinary NGAL, Robo4 levels were also significantly different (AKI: 6335 ng/ml, IQR 5025–7771, n = 6; vs non-AKI: 3723 ng/ml, IQR 2840–5028, p = 0041). When patients were grouped according to AKIN guidelines, there was a significant difference (p = 0.003) between AKI (6546 pg/ml, IQR 5025–8079; n = 6) and non-AKI patients (3727 pg/ml, IQR 1962–3727; n = 26; Fig. 3).

For comparison, when peak levels of vWF or Ang-2 (see Table 3) were also grouped according to NGAL and AKIN criteria; the levels of vWF (2 h) in the >150 ng/ml plasma NGAL group (163.3±48.8%) were significantly higher (p = 0.0042) than the <150 ng/ml NGAL group (109.9±48.9%). By contrast, vWF levels were not significantly different when grouped according to the AKIN definition (data not shown). The reverse was true for 24 h Ang-2 levels, with significantly higher (p = 0.003) levels in the AKI group, defined using the AKIN definition (9.81 pg/ml, IQR 8.45–12.2 vs. 6.34 pg/ml, IQR 4.49–8.9) but not the plasma NGAL cut-off definition (data not shown).

Finally, neither duration of mechanical ventilation (16.5 h, IQR 11.5–2.1) nor PF ratio (313.7±102.3) associated significantly with Robo4 levels (data not shown). Likewise, neither pre- nor post-operative levels of Ang-2 were associated with Robo4 (data not shown). By contrast, pre-operative Robo4 and vWF levels were significantly, positively correlated (r = 0.561, r^2^ = 0.314, p = 0.0008); as were levels of these biomarkers at 24 h (r = 0.4895, r^2^ = 0.2396, p = 0.0045), but not those measured 2 h post-operatively.

## Discussion

We have shown for the first time that the membrane-associated regulatory protein of endothelial activation, Robo4, is detectable in plasma of patients undergoing cardiac surgery requiring CPB; and that levels were raised, 2 h post-operatively, and returned to baseline by 24 h. Whilst pre-operative Robo4 levels correlated with pre-operative risk (EUROscore) assessment, there was no significant difference between groups when patients were classified as ‘high’ or ‘low’ risk. By contrast, increased plasma Robo4, measured 2 h post-operatively, did not correlate with operative variables; post-operative outcomes (level 3 care, ICU and hospital LOS); indices of pulmonary dysfunction (PF ratio, duration of mechanical ventilation), or raised levels of conventional plasma endothelial markers (Ang2 and vWF), also measured at 2 h. Whilst no associations with pro-inflammatory (IL-6 or -8) cytokines were demonstrated, there was a positive correlation with levels of the anti-inflammatory cytokine, IL-10. Raised post-operative plasma Robo4 levels correlated significantly and positively with plasma and urine NGAL levels; associations maintained when patients were divided into those with and without AKI, using an NGAL cut-off level of 150 ng/ml and also AKIN classification. These novel findings, suggesting a possible association between raised plasma Robo4 levels after surgery and AKI, might indicate a useful, subclinical diagnostic marker of cardiac surgery associated (CSA)-AKI; and also could point to a possible therapeutic target to limit this complication.

We (and others) have hypothesised that Robo4 could be a clinically useful marker of endothelial dysfunction. vWF is considered the canonical endothelial biomarker. In the current study the increased levels of vWF detected were similar to those reported previously, with an increase at 2 h and remaining elevated at 24 h [9]; decreasing by day 3 [10]. Ang-2 is also a well-characterised endothelial biomarker, particularly of barrier function, high levels of which have been reported 24 h after CPB with levels rising from 2.6 pre to 7.3 ng/ml 24 h post-operatively [12]; levels very similar to those reported here (2.3 up to 7.3 ng/ml).

Ang-2 and vWF profiles in this study match those previously reported; but differed from Robo4 which displayed an early increase (2 h) and a return to basal levels by 24 h. Whilst limitation of sample availability precluded a study in all patients at more time points, a pilot study in 3 patients suggested that Robo4 was elevated to similar levels immediately after surgery and at 2 h, decreasing to basal levels by 4 h; implying a rapid rise and fall in plasma levels. It is possible that this points to rapid cleavage, followed by clearance of the protein. Raised Robo4 levels 2 h after surgery requiring CPB did not correlate with post-operative vWF and Ang-2 levels at this time point, but this does not necessarily imply that Robo4 is not endothelial-derived. Rapid removal of Robo4 from the cell surface could possibly act as a first, fast response to trauma; i.e. to release the protective check on barrier function. By contrast, more sustained release of vWF or Ang2 from endothelial Weibel-Palade bodies is characteristic of the ongoing response to trauma/inflammation. It is also possible that the association between pre-operative levels of vWF and Robo4 represent continuous, basal turnover of endothelial-associated molecules. We have unpublished data showing Robo4 was also detectable in plasma from healthy control volunteers, at levels similar to those seen in patients’ pre-surgery with CPB, also perhaps suggestive of on-going Robo4 turnover.

A greater understanding of the mechanism(s) responsible for increased Robo4 plasma levels, which we are assuming results from cleavage, could help to shed light on the significance of plasma Robo4. However, to date, little is known concerning the release of the ectodomain of any Robo receptor. A recent study investigated the juxtamembrane domain of Robo1 and showed that unfolding of the extracellular juxtamembrane linker region was related to shedding [32]; and others have suggested that the matrix metalloproteinase, ADAM10, is involved in Robo1 shedding [33].

Whatever the mechanism(s) responsible for an increase in plasma Robo4; what might the other associations and indeed the lack of associations, reported in our study point to concerning the functional significance of Robo4? It is perhaps surprising that duration from start of anaesthesia to end of CPB did not correlate with Robo4 levels, given the injurious factors associated with surgery together with CPB including: activation of blood during exposure to extra-corporeal circuitry, relative hypo-perfusion, myocardial and pulmonary ischemia and operative tissue injury [34]. However, it is of note that unlike our previous study in this clinical scenario [31], the time recorded was not strictly analogous to duration of CPB; although it encompassed this parameter and was employed because of ease of extraction from clinical databases. We acknowledge this as a limitation to our study. By contrast, why Robo4 levels positively associated with IL-10 but not the other cytokines measured, is also unclear. Raised plasma levels of IL-10, 24 h post-operatively, are predictive of complications after CPB [35], but whether IL-10 could be responsible for Robo4 shedding is unknown.

Finally, it would appear that the kidney is sensitive to subtle changes in Robo4 levels (reflected in associations with AKI); whereas, the lung is not (given the apparent lack of association with indices of pulmonary dysfunction). Certainly in terms of the perceived problems associated with this patient group, the need to understand and to be able to predict and prevent AKI is possibly more pressing clinically; not least, because the mean PF ratio for this, albeit small number of patients, was less than that required to fulfil the criteria for acute lung injury [36]. By contrast, AKI assumes prognostic significance in this patient group, as we and others have shown [4], [5]. Moreover, a requirement for biomarkers that can be used to make subclinical diagnoses of CSA-AKI and thus allow interventions before acute tubular necrosis has already occurred, are of importance in this clinical specialty [4], [5]; and beyond, not least, given the understanding that AKI and chronic kidney disease are interconnected syndromes [37].

We have shown a significantly increased level of plasma Robo4 in patients deemed to have AKI, not only using the more conservative AKIN classification based on serum creatinine levels, but also employing the novel AKI plasma and urine biomarker, NGAL. Measurement of NGAL facilitates early detection of AKI, 2 h post-surgery; a time which also corresponds to peak plasma Robo4 levels. Our novel finding suggests that Robo4 merits further consideration as a potential early indicator of AKI. Further support that Robo4 might not be just a coincidental biomarker of AKI but point to a functional role, is acknowledged through its endothelial barrier regulatory function [38]. Given that one of the few currently effective renoprotective agents, nesiritide, protects against endothelial barrier dysfunction [39]; also points to endothelial protective strategies to limit CSA-AKI. In conclusion, Robo4 merits further investigation both as an endothelial biomarker and as a target for new pharmacological therapeutic interventions.
